# Good Amyloid, Bad Amyloid—What’s the Difference?

**DOI:** 10.1371/journal.pbio.1002362

**Published:** 2016-01-26

**Authors:** Roland G. Roberts

**Affiliations:** Public Library of Science, Cambridge, United Kingdom

## Abstract

Why do some amyloids cause serious neurodegenerative diseases, while others have important biological functions? A new study of the functional amyloid Orb2 suggests that it's all about speed. Read the Research Article.

Amyloids have had some bad press. From Alzheimer to Parkinson, Huntington to Creutzfeldt-Jakob, amyloids are best known for their role in causing some very serious human diseases. The usual story is that the structure of a perfectly respectable, neatly folded (and usually soluble) protein is disrupted in some way, making it refold into a new “cross-beta” structure that predisposes multiple copies of that protein to tile together into a massive insoluble fibrous deposit. The trigger may be a mutation, an unusual cleavage pattern, a post-translational modification, or even (in the case of a class of amyloids called prions) infection by a ready-formed amyloid, but the end point tends to be the same—insoluble protein, cognitive problems, and eventual neurodegeneration.

Research has understandably been focused on the pathogenic amyloids that are involved in human neurological diseases, but several points have recently become clear. The first is that despite their suspicious presence at the scene of the crime, the insoluble deposits of amyloid may not themselves be the culprits and that instead it’s the smaller “oligomeric” assemblages or even the monomers of amyloidogenic protein (en route to fiber formation) that may cause the real damage. And the second is that not all amyloids are bad—some are biologically useful and have been selected for their beneficial function during evolutionary history.

One such “good” amyloid is generated by an RNA-binding protein called cytoplasmic polyadenylation element binding protein (CPEB). As well as containing RNA-binding motifs that help it regulate the translation of a subset of cellular mRNAs, some forms of CPEB (including the fruit fly version, Orb2) have a domain that can flip into an amyloid-forming alternative structure. Studies in the fruit fly *Drosophila*, the sea slug *Aplysia*, and mice have shown that the formation of amyloids by CPEB/Orb2 is important for the consolidation of memory, in a way that’s independent of the protein’s RNA-binding day job.

But what is the crucial difference between a well-behaved, functional amyloid and a lethally destructive amyloid? A new study by Rubén Hervás, Liying Li, Kausik Si, Mariano Carrión-Vázquez, and colleagues, just published in *PLOS Biology*, may have part of the answer.

The authors focus on fruit fly Orb2 as an example of a “good” amyloid. First, they confirm that the Orb2’s amyloid-forming domain (the prion-like domain, or PLD) behaves broadly like other (“bad”) amyloids. They find that when freshly made, the Orb2 PLD is soluble and mostly α-helical in structure but that the protein soon spontaneously and efficiently converts to a largely β-sheet structure, forming first oligomers and then the typical insoluble amyloid fibers (Fig 1).

**Fig 1:**
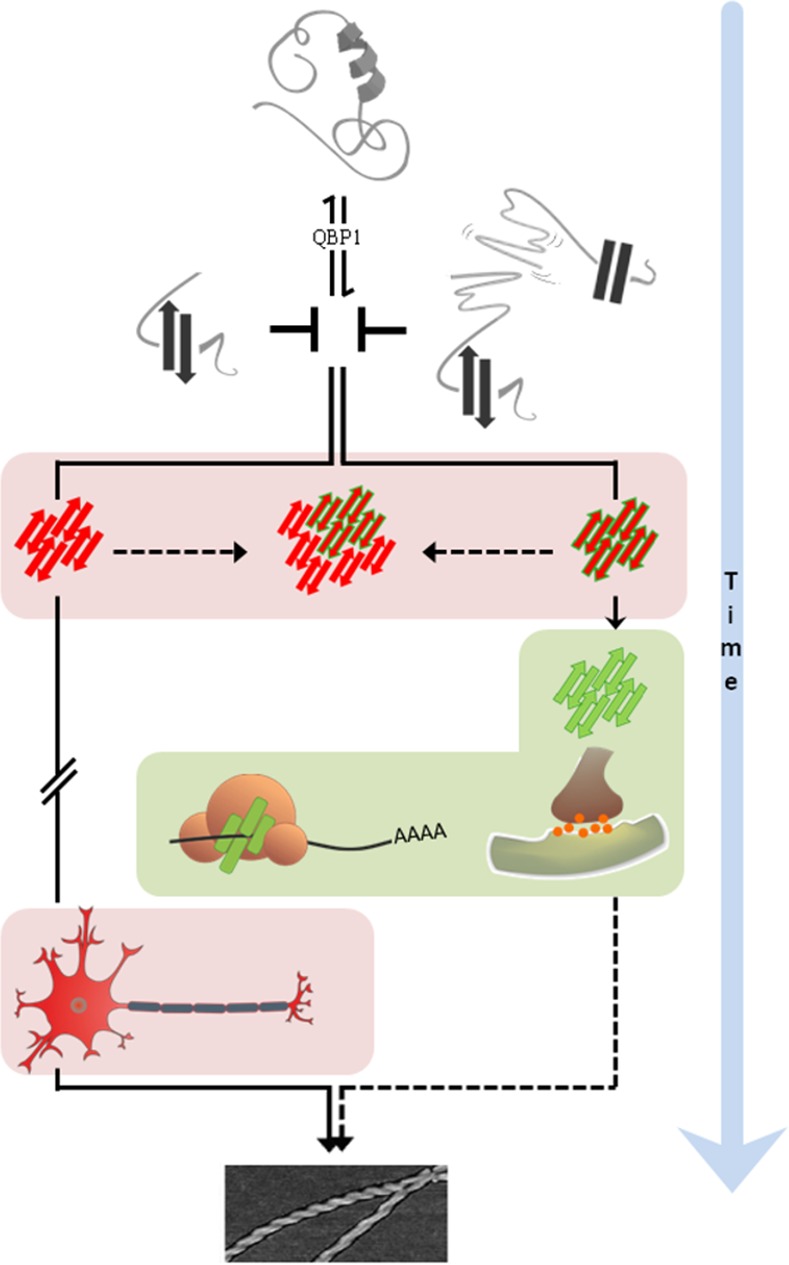
“Good” and “bad” amyloids. Both pathological (red) and functional (green) amyloids can be inhibited at the beginning of the cascade by a common peptide (QBP1), and they both form toxic oligomers. However, the life span of the pathological species is much longer. Still, these two types of oligomers share enough similarities to form hybrid species, suggesting that the pathological amyloids may be sequestering the functional ones, thereby impairing their function. *Image credit*: *doi*:*10*.*1371/journal*.*pbio*.*1002361*.

Further experiments show that Orb2 PLD amyloid formation also seems to have the infectious self-propagating properties of prions and that like many other prions, one half of the PLD is responsible for initiating protein clumping, while the other half directs recruitment into already-formed aggregates. By stretching individual molecules, the authors were also able to show that the PLD has the wide structural variation characteristic of other amyloid-forming proteins.

So to all intents and purposes, these and other observations confirm that Orb2 is a card-carrying amyloidogenic protein. But what allows it to function happily as an amyloid in the fly nervous system without causing a catastrophic neurodegenerative disease?

One possibility might be that the oligomers, increasingly fingered as the pathogenic agents, differ between “good” and “bad” amyloids. The authors had already noticed that Orb2 amyloids form surprisingly quickly and propagate with unusual stability as prions in yeast. To investigate this further, they directly compared the behavior of Orb2 and the Alzheimer amyloidogenic peptide Aβ42 in the test tube. Strikingly, Orb2 started to change conformation within minutes, while Aβ42 was stable for days (Fig 1).

What are the consequences of this for cells? The authors used chemical inhibitors to trap Orb2 in either the oligomeric form or the fibrous form and then injected them into cultured cells. As has been found for other amyloids, only the trapped oligomeric form of Orb2 (now made stable over time) caused widespread cell death.

Thus it seems that at least one of the differences between “good” and “bad” amyloids might lie simply in an aspect of their kinetics, namely the time dwelt in the oligomeric intermediate form. To test this, the authors swapped the PLD of Orb2 with the polyglutamine tract from human mutant huntingtin protein—a well-studied pathogenic amyloid that causes Huntington disease. Strikingly, huntingtin containing Orb2’s PLD rapidly converted into a nontoxic amyloid, while Orb2 containing huntingtin’s polyglutamine tract converted much more slowly via a highly toxic oligomeric state. Clearly, the kinetics and toxicity (fast and safe versus slow and deadly) are intrinsic properties of the respective amyloidogenic protein regions.

Given that “good” and “bad” amyloids behave so similarly in many respects, is there the possibility that they might influence each other when both are present? The authors tested this by coexpressing Orb2 and polyglutamine-containing huntingtin, finding that the toxic huntingtin coaggregates with Orb2. They speculate that toxic amyloids might have the ability to directly interfere with memory consolidation by mopping up functional amyloids of the CPEB/Orb2 family.

The authors further showed that a peptide known to block the formation of pathogenic amyloids (QBP1) also blocks the formation of Orb2 amyloid (Fig 1), presumably because both types of amyloid are conformationally similar. But does this mean that while it can block the ill effects of “bad” amyloids, QBP1 can also interfere with the biological functions of “good” amyloids?

To address this, the authors engineered fruit flies so that they make QBP1—or a nonfunctional scrambled version—in all of their neurons. They then subjected the flies to a memory test in which males learn that it’s not worth investing effort in courting unreceptive females. Neither QBP1 nor the scrambled version affected short-term memory, but when the males were tested 24 hours later, the males whose neurons contained QBP1 had forgotten the crushing disappointment of their previous amorous attempts, indicating a failure of long-term memory. The inference is that a peptide known to inhibit formation of “bad” amyloid can also inhibit important biological processes (such as memory consolidation) that depend on “good” amyloid, raising questions about the potential side effects of inhibiting amyloid formation in human patients.

Conversely, can blocking memory consolidation be beneficial? QBP1 or its chemical analogues may prove to be useful compounds to prevent the consolidation of traumatic or toxic memories in post-traumatic stress disorder and related diseases.

This study shows that the functional amyloid Orb2 shares a wide suite of properties with its pathogenic cousins, including conversion to a structurally characteristic insoluble fibrous deposit via a toxic oligomeric intermediate. However, a crucial difference seems to be the fleeting nature of Orb2’s toxic form. Presumably evolution, while honing the functional amyloid for a biological purpose—the consolidation of memories—has selected for rapid kinetics that cut to the chase and minimize the chances of lasting damage. Pathogenic amyloids have not had that luxury.

## Abbreviations

CPEB: cytoplasmic polyadenylation element binding protein
PLD: prion-like domain
